# Consumers’ Exposure to Nutrition and Health Claims on Pre-Packed Foods: Use of Sales Weighting for Assessing the Food Supply in Slovenia

**DOI:** 10.3390/nu7115474

**Published:** 2015-11-12

**Authors:** Igor Pravst, Anita Kušar

**Affiliations:** Nutrition Institute, Tržaška cesta 40, Ljubljana 1000, Slovenia; anita.kusar@nutris.org

**Keywords:** food labeling, health claims, nutrition claims, nutrition declaration, food supply

## Abstract

Insights into the use of health-related information on foods are important for planning studies about the effects of such information on the consumer’s understanding, purchasing, and consumption of foods, and also support further food policy decisions. We tested the use of sales data for weighting consumers’ exposure to health-related labeling information in the Slovenian food supply. Food labeling data were collected from 6342 pre-packed foods available in four different food stores in Slovenia. Consumers’ exposure was calculated as the percentage of available food products with particular food information in the food category. In addition, 12-month sales data were used to calculate sales weighted exposure as a percentage of sold food products with certain food information in the food category. The consumer’s in-store and sales-weighted exposure to nutrition claims was 37% and 45%, respectively. Exposure to health claims was much lower (13%, 11% when sales-weighted). Health claims were mainly found in the form of general non-specific claims or function claims, while children’s development and reduction of disease risk claims were present on only 0.1% and 0.2% of the investigated foods, respectively. Sales data were found very useful for establishing a reliable estimation of consumers’ exposure to information provided on food labels. The high penetration of health-related information on food labels indicates that careful regulation of this area is appropriate. Further studies should focus on assessing the nutritional quality of foods labeled with nutrition and health claims, and understanding the importance of such labeling techniques for consumers’ food preferences and choices.

## 1. Introduction

Health-related information on food labels might play a role in influencing their purchase decisions [1,2,3,4,5,6,7,8] and is, therefore, carefully regulated in most developed countries to make sure consumers are given non-misleading information. The use of nutrition and health claims was harmonized in the European Union (EU) with Regulation (EC) 1924/2006 on nutrition and health claims (NHCR; the regulation); only pre-defined nutritional claims are allowed and all specific health claims need to be substantiated by generally-accepted scientific data, non-misleading, and pre-approved [9]. The types of claims that can be made on foods in the EU are presented in Table 1.

Implementation of the NHCR has involved a steep learning curve for different stakeholders, including authorities and the industry [10,11], and many foods on the market are being reformulated and/or relabeled. The importance of a varied and balanced diet is well established and it is known that individual foods hold relative importance in the context of one’s overall diet. To avoid a situation where claims would mask the overall nutritional status of a food product and mislead consumers when trying to make healthy choices, regulatory limits on the nutritional composition of foods carrying claims (nutrient profiles) should have been introduced in the EU in 2009, but this part of the legislation has not yet been implemented [12]. Particularly in the absence of such measures limiting the use of health-related information on food labels, it is vital to investigate the penetration of claims on food labels, and to identify commonly-used claims and food categories where such claims are used. In fact, the danger that a nutrition or health claim might encourage excessive consumption of specific foods was identified as a relevant food policy issue when the regulation was accepted [9]. This resulted in a demand for a mandatory assessment of the NHCR, particularly about the evolution of the market in foods with respect to the use of nutrition or health claims (Art. 27 of the NHCR).

**Table 1:** Categorization of nutrition and health claims in the European Union according to the NHCR.

Reference to the NHCR	Claim Type	Definition	Examples of Wordings
Art. 8	Nutrition claims	Claims referring to particular beneficial nutritional properties of the food	High in vitamin D
Art. 13	Function claims (FC)	Claims referring to (a) the role of a nutrient or other substance in growth, development and the functions of the body; (b) psychological and behavioral functions; or (c) slimming or weight-control or a reduction in the sense of hunger or an increase in the sense of satiety or to the reduction of the available energy from the diet	Vitamin D contributes to the maintenance of normal bones.
Art. 14(1)a	Reduction of disease risk claim (RDRC)	Claims that state, suggest or imply that the consumption of a food (constituent/category) significantly reduces a risk factor in the development of a human disease	Vitamin D helps to reduce the risk of falling associated with postural instability and muscle weakness. Falling is a risk factor for bone fractures among men and women 60 years of age and older.
Art. 14(1)b	Children's development and health claims (CDHC)	No specific definition in the regulation	Vitamin D is needed for normal growth and development of bone in children.
Art. 10(3) Art. 1(3)	General non-specific health claim	References to general, non-specific benefits of the nutrient or food for overall good health or health-related well-being. Such claims can also include trademarks and brand names.	Bone health support

Such an assessment requires the food categories with the highest penetration of nutrition, health claims, and the types of claims most commonly used on food labels to be identified. While some studies are available from jurisdictions with a longer tradition in the use of health claims on foods (for example from Canada [13,14], the USA [15,16], Australia, and New Zealand [17,18,19,20,21], data from EU member states is limited. In a FLABEL (Food Labeling to Advance Better Education for Life) study from 2009, the penetration of nutrition and health claims was reported [22], while the study did not analyze the types of claims. Another in-depth across-the-market analysis was conducted in Ireland where 47% and 18% of surveyed pre-packed foods were labeled with nutrition and health claims, respectively [23]; data were collected on 1880 commonly eaten foods found in four major retailer stores. The study was performed in 2007 just after the new regulation had been accepted and will be an important source of information for assessing changes in the food market. In addition, analysis of products available in *on-line* store of a major UK food retailer (2011/2012 data) was also reported very recently [24].

It should be noted that all previous studies focused on consumers’ exposure to claims on available foods in the food supply, without considering that the importance of different products can vary greatly due to different market shares. Such differences can be taken into account by weighting using sales data. While such a strategy was already successfully used in other types of evaluations, for example in the assessment of the content of sodium [25] and iodine [26] in the food supply, to our knowledge such an approach has not yet been used to provide insights on consumers’ exposure to health-related food labeling information.

Investigation of the food supply is very challenging due to the diversity of available foods and retail environments. A step-wise approach was proposed for the selection of foods and retail outlets; optimal monitoring would ideally include all foods in all retail outlets in the jurisdiction [27], although in most cases this is impossible to achieve. Some global initiatives address the harmonization of data collection, such as the International Network for Food and Obesity Research, Monitoring and Action Support (INFORMAS) [27] and the Global Food Monitoring Group [28], with the latter largely concentrating on the nutritional composition of foods. A huge volume of data is collected/generated in such studies, particularly when studies target a wide selection of food categories. Assuring the representative nature of the food sample, while remaining capable of dealing with the data collection and evaluation, is, therefore, a key goal of all such studies.

Although Slovenia joined the EU in 2004, a few years before the NHCR was introduced, accession to the union affected its food law considerably. Further, contrary to some other EU countries where health claims were allowed on foods also prior to the NHCR being introduced in 2007, this was not the case in Slovenia where such claims were interpreted as medicinal claims and not tolerated on foods. Due to these, and several other, factors, including cultural ones, notable differences exist in the food supply between different EU countries, as well as in dietary patterns in various populations.

A specific research project was launched in Slovenia (see Acknowledgments) to address the above-mentioned challenges. The objective of this study was to investigate consumers’ exposure to nutrition and health claims on pre-packed foods in the food supply in Slovenia also using an innovative sales-weighting approach. The study was planned to provide information on which food categories have the highest prevalence of health-related information on food labels, and which are the most common nutrients and health relationships mentioned in the claims.

## 2. Experimental Section

### 2.1. Collection of Data

Using the food labeling information we compiled a database of pre-packed foods on the market. The full sample included all foods (*n* = 6341) within selected food categories available in selected grocery stores at the time of sampling (January 2011).

Sampling was done in four grocery stores in the capital, Ljubljana: one large supermarket (LAS), two neighborhood stores (NS1, NS2), and one discounter store (DIS). To ensure high representativeness of the sample, we selected grocery stores of retailers with accessible nation-wide store networks, and with the biggest market shares. The retailers included accounted for 66% of the total national market share in terms of sales value, and operated in all parts of the country.

The selection of food categories was made according to Lalor *et al.* [23] with the addition of the following categories where we expected a notable presence of nutrition and health claims: processed seafood, ready-made products, vegetable oils, and plant-based imitations of milk and yoghurt. The sample of foods in this study, therefore, does not include non-pre-packed foods (including fresh fruit and vegetables), and also certain categories of pre-packed foods, *i.e.*, food supplements, baby foods, alcoholic drinks, confectionery, unprocessed cereals, and snacks. All products were also classified according to the FOODEX2 classification system [29].

With the selected retailers’ approval, data were collected in the selected stores by two researchers and five trained food technology students. A special computer application was developed to support the efficient inputting of food labeling data into an SQL database and to avoid duplicate work. First, the European/International Article Number (EAN barcode) was scanned for each investigated product. A computer application was then used to check if the product had already been included in the database. If this was the case, the scanned information was inserted into the database to provide data about the availability of a particular food in a particular grocery store. If a product was not yet in the database, the following information was extracted from the food label: product brand and name, producer, nutrition declaration (data about nutritional composition), nutrition claim(s), health claim(s), nutrients or other substances related with nutrition/health claims, and information about the presence of health and other symbols.

**Figure 1:**
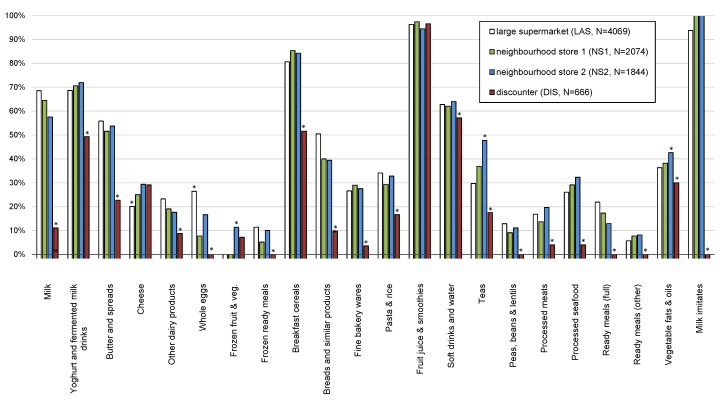
Prevalence of nutrition and/or health claims on pre-packed foods in the large supermarket (LAS), two neighborhood stores (NS) and the discounter (DIS) (Notes: statistically significant difference in the prevalence in comparison to other stores (dual-mode criteria). See Supplementary Table S2).

After collecting the data, the retailers were asked to provide 12-month, country-wide sales volume data for each product included in the database (January 2011–December 2011). Ensuring proper data handling, we were able to obtain sales data from retailers covering over 62% of the national market. Sales data were available for 5104 foods in the above-mentioned database (80.5%) and this sample was used for further analyses (Table 2, Table 3 and Table 4 and Supplementary Tables S1, S3, and S4), except for comparisons between different store types (Figure 1, supplementary Table S2) which was done on the full sample of foods. One retailer (DIS) decided not to share its sales data due to its internal company policies. Unfortunately, such a policy is in place in all discounters in Slovenia. It should be noted that the sales data retailers provided referred to the complete national market and presented food product sales for the same year in which the above-mentioned food composition database was compiled. This was arranged on the condition that the results would not reveal the sales data of any particular retailer/product. The sales data were given in universal form and included the EAN barcode, description of the product, number of products sold per year and the quantity of food (kg/L) per packaging. The matching of foods between the databases was performed using EAN barcodes.

**Table 2:** Consumer’s exposure to nutrition and health claims and symbols on pre-packed foods.

Food Category	*N*	Nutrition Claim(s)		Health Claim (s)		Nutrition and/or Health Claim (s)		Health Symbol		Nutrition Declaration	
		SCE	SWE	SCE	SWE	SCE	SWE	SCE	SWE	SCE	SWE
Milk	53	53%	68%	11%	3%	53%	68%	2%	1%	94%	97%
Yoghurt and fermented milk drinks	294	65%	64%	51%	46%	66%	65%	14%	20%	94%	98%
Butter and spreads	196	51%	49%	18%	26%	56%	60%	0%	0%	79%	71%
Cheese	335	21%	30%	2%	10%	21%	30%	1%	8%	45%	72%
Other dairy products	138	20%	10%	4%	2%	20%	10%	0%	0%	70%	63%
Whole eggs	42	19%	1%	5%	1%	24%	2%	0%	0%	14%	6%
Frozen fruit & vegetables	111	4%	1%	1%	0%	4%	1%	0%	0%	90%	90%
Frozen ready meals	239	10%	6%	2%	1%	11%	6%	0%	0%	89%	95%
Breakfast cereals	276	79%	83%	31%	19%	80%	84%	5%	7%	96%	97%
Breads and similar products	297	47%	10%	8%	3%	47%	10%	2%	1%	59%	26%
Fine bakery wares (biscuits)	323	26%	22%	5%	2%	26%	22%	0%	0%	53%	51%
Pasta & rice	436	27%	11%	11%	13%	31%	23%	3%	1%	86%	80%
Fruit juice & smoothies	240	95%	99%	15%	14%	95%	99%	0%	0%	95%	100%
Soft drinks and water	459	63%	41%	9%	10%	63%	42%	0%	0%	83%	62%
Teas	362	21%	33%	19%	5%	31%	34%	0%	0%	13%	18%
Peas, beans & lentils	97	10%	2%	6%	2%	11%	3%	4%	2%	56%	60%
Processed meats	429	15%	8%	7%	4%	17%	11%	3%	3%	45%	62%
Processed seafood	237	25%	35%	3%	1%	26%	35%	0%	0%	49%	70%
Ready meals – full meal	96	19%	10%	0%	0%	19%	10%	0%	0%	89%	84%
Ready meals – other	133	6%	3%	2%	1%	6%	3%	0%	0%	59%	64%
Vegetable fats and oils	194	33%	12%	20%	6%	38%	14%	10%	4%	56%	22%
Milk imitates	35	94%	98%	6%	13%	94%	98%	0%	0%	100%	100%
Yoghurt imitates	27	81%	82%	63%	72%	81%	82%	0%	0%	100%	100%
Chewing gum	55	0%	0%	9%	3%	9%	3%	0%	0%	71%	80%
TOTAL	5104	37%		13%		39%		2%		67%	
TOTAL (excluding eggs and chewing gums)	5007	37%	45%	13%	11%	39%	46%	2%	2%	67%	72%

Notes: SCE—store exposure: percentage of available food products with particular food information in the food category; SWE—sales weighted exposure: percentage of sold food products with particular food information in the food category (calculated per kg/L, except in eggs/chewing gums where the calculation is per piece). Total SWE values are calculated for food categories for which sales data for kg/L of food were available (all foods except eggs and chewing gums). See supplementary Table S1.

**Table 3:** Frequencies of common nutrients and other substances in nutrition claims per food category.

Food Category	Energy		Protein		Sugar		Fat		Fiber		Sodium/Salt		Mineral (s)		Vitamin (s)	
	SCE	SWE	SCE	SWE	SCE	SWE	SCE	SWE	SCE	SWE	SCE	SWE	SCE	SWE	SCE	SWE
Milk			2%	1%			2%	1%					47%	65%	6%	3%
Yoghurt and fermented milk drinks	0%	1%	3%	2%	3%	1%	23%	33%	3%	6%			13%	9%	8%	8%
Butter and spreads			1%	0%			18%	9%	5%	0%	1%	0%	3%	1%	21%	44%
Cheese			0%	0%			6%	24%	0%	0%			12%	11%	3%	3%
Other dairy products			1%	0%	1%	1%	2%	1%					15%	7%	8%	5%
Whole eggs													7%	0%	10%	0%
Frozen fruit and vegetables			1%	0%	3%	1%							1%	0%		
Frozen ready meals			4%	1%			1%	0%	3%	1%			2%	0%	2%	0%
Breakfast cereals					15%	5%	8%	3%	38%	32%	8%	1%	37%	43%	47%	66%
Breads and similar products	0%	0%			1%	0%	5%	1%	33%	7%	1%	0%	4%	2%	1%	1%
Fine bakery wares (biscuits)	0%	0%	1%	1%	3%	1%			14%	11%	2%	0%	6%	14%	3%	1%
Pasta & rice							4%	0%	10%	3%			7%	6%	3%	4%
Fruit juice and smoothies	0%	0%			38%	34%	1%	0%	0%	0%	0%	0%	8%	13%	29%	23%
Soft drinks and water	20%	15%			4%	1%			2%	0%	3%	5%	9%	11%	23%	10%
Teas					0%	0%							1%	0%	4%	8%
Peas, beans, and lentils			2%	0%					7%	2%			5%	0%	5%	0%
Processed meats			1%	0%			4%	4%	1%	0%	0%	0%			0%	0%
Processed seafood	1%	0%	2%	0%	1%	0%							5%	15%	5%	14%
Ready meals—full meal	1%	1%			1%	0%	6%	7%	2%	3%			2%	0%	2%	0%
Ready meals—other			1%	0%					4%	3%						
Vegetable fats and oils													1%	0%	19%	10%
Milk imitates			20%	31%	6%	13%					3%	6%	34%	55%	9%	1%
Yoghurt imitates			19%	20%	4%	1%	22%	17%	4%	7%	4%	11%	41%	27%	19%	16%
Chewing gum																
TOTAL	2%		1%		4%		4%		7%		1%		8%		10%	

Notes: SCE—store exposure: percentage of available food products with particular food information in the food category; SWE—sales weighted exposure: percentage of sold food products with particular food information in the food category (calculated per kg/L, except in eggs/chewing gums where the calculation is per piece). Sample size is provided in Table 2. See also supplementary Table S3.

**Table 4:** Proportion of pre-packed foods (per food category) with different categories of health claims.

		**Health claim category**					**Health relationship (classification according to ICF/WHO body functions)**											
**Food category**	***N***	**General non-specific health claims**	**Function claims**	**Children's development and health**	**Reduction of disease risk claims**		**Mental functions (b1)**	**Cardiovascular system functions (b410-b429)**	**Haematological system functions (b430)**	- Blood cholesterol levels (b4302)	**Immunological system functions (b435)**	**Digestive system functions (b510-b539)**	- Glycaemic response (b5152)	- Weight maintenancefunctions (b530)	**Metabolic functions (b540-b559)**	**Bones (b720) & teeth functions (b5101)**	**Muscle functions (b730-b749)**	**Functions of the skin, hair, nails (b810-b869)**
Milk	53		7.5%	1.9%	3.8%			1.9%	1.9%	1.9%	1.9%	1.9%				1.9%		
Yoghurt and fermented milk drinks	294	35.4%	24.1%		1.0%		0.3%		1.0%	1.0%	7.1%	12.2%		2.7%		2.4%		
Butter and spreads	196	13.8%	4.6%		0.5%		1.0%	2.0%	2.0%	2.0%		0.5%						
Cheese	335	0.9%	0.9%								0.6%	0.9%						
Other dairy products	138		3.6%													3.6%		
Whole eggs	42		4.8%					4.8%										
Frozen fruit & vegetables	111	0.9%																
Frozen ready meals	239	2.1%																
Breakfast cereals	276	10.1%	19.6%	1.1%	0.4%		5.8%	2.5%	2.9%	2.5%	2.5%	7.2%	3.3%	7.2%	1.1%	2.5%	0.7%	0.4%
Breads and similar products	297	4.0%	4.0%	0.3%	0.7%				1.0%	1.0%	0.3%	2.4%	1.3%	1.3%	0.3%	0.7%	0.3%	
Fine bakery wares (biscuits)	323	1.5%	4.3%				0.3%				0.9%	3.1%	0.9%	1.5%	0.9%			0.9%
Pasta & rice	436	0.9%	8.0%				0.2%		0.2%	0.2%	0.2%	5.0%	4.1%	3.0%		0.2%	0.2%	
Fruit juice & smoothies	240	6.3%	9.2%	0.4%			1.7%	1.3%			3.3%	1.3%		1.3%				0.4%
Soft drinks and water	459	3.7%	7.6%				2.4%				1.7%	2.6%	1.1%	2.4%	1.7%	2.0%	0.7%	
Teas	362	3.0%	17.1%				3.9%		1.1%		9.1%	6.4%		1.4%	0.3%			0.8%
Peas, beans & lentils	97	1.0%	1.0%						1.0%		1.0%	1.0%	1.0%		1.0%		1.0%	
Processed meats	429	4.9%	0.2%								0.2%			0.2%				
Processed seafood	237	2.1%	0.8%					0.8%										
Ready meals - full meal	96																	
Ready meals - other	133	0.8%	2.3%						0.8%	0.8%	0.8%	0.8%		0.8%				
Vegetable fats and oils	194	5.2%	7.7%		1.5%		1.0%	5.7%	0.5%	0.5%	2.1%	1.5%			0.5%			0.5%
Milk imitates	35	5.7%																
Yoghurt imitates	27	55.6%	7.4%											7.4%				
Chewing gum	55		9.1%								1.8%					7.3%		
TOTAL	5104	5.6%	7.0%	0.1%	0.2%		1.0%	0.6%	0.5%	0.4%	1.8%	2.8%	0.8%	1.4%	0.4%	0.7%	0.2%	0.2%

Notes: Also see supplementary Table S4.

### 2.2. Consumers’ Exposure to Nutrition/Health Claims and Health Symbols on Pre-Packed Foods

Percentage of available food products with specific food information in the food category (SCE—store exposure) was calculated within selected food categories. SCE values (%) present the proportion of available foods labeled with specific information (*i.e.*, nutrition/health claim/symbol) within all foods available in the (sub) category.

Percentage of sold food products with specific food information in the food category (SWE—sales weighted exposure) was also calculated for selected food categories. Since several of the same food products were available in different stores, the sales data provided by the retailers were combined to calculate the number of products sold per year for each food product in the database. Using data on the content of food per package, we calculated the amounts of products sold per year (for all available products within the food category and for all available products labeled with specific food information). SWE values (%) therefore present the proportion of amounts of sold foods labeled with specific food information (*i.e.*, nutrition/health claim/symbol) within all foods sold in the (sub) category. For most food categories all calculations were done per kg/L, except for eggs and chewing gums, where calculations are per piece.

Identification of the health claims was performed according to definitions in the NHCR (Table 1). Claims (including pictorial, graphic or symbolic representation in any form) stating, suggesting or implying the existence of a relationship between a food and health were considered as health claims. Health claims were categorized as: (a) function claims (as defined in Arts. 13 and 13.5 of the regulation); (b) reduction of disease risk claim (Art. 14(1)a); (c) claims referring to children’s development and health (Art. 14(1)b); and (d) general non-specific health claims (as defined in Art. 10(3); including trademarks and brand names which may be construed as a claim). In addition, all claims were classified according to subject nutrient/substance (energy and macronutrients, minerals, vitamins, other substances) and subject-health relationship. Classification of the health relationships was made according to the WHO International Classification of Functioning, Disability and Health (ICF Body functions) [30].

### 2.3. Data Validation

The accuracy of the data collection and coding was assured using a confirmation procedure. The data collection was done in pairs directly in the store. After the data for a specific food were typed into the software, they were re-checked by a second researcher. Data on all nutrition and health claims were collected. If the data-entry staff were unsure whether a specific claim is a nutrition or health claim, the claim was inserted into the database and the issue was resolved later on with the rest of the research team. Classification of the foods and claims was also done using a confirmation procedure. After all the classifications had been made (A.K., Ž.K.), the classifications were double-checked by another researcher (I.P.). Open issues were discussed within the research team.

### 2.4. Statistical Analyses

Statistical analyses were performed using Microsoft Excel 2013 (Microsoft Corporation, Redmond, DC, USA). Food labeling information was collected for all available food products, therefore both SCE and SWE values are given as exact values and no standard deviations are presented. A comparison of the prevalence of specific food information between different stores (Figure 1, Table S2) was made within selected food categories employing dual-mode hypothesis. We calculated the difference between the SWE value (in a selected grocery store, primary mode) and the average of SWE values for other stores (second mode). The difference was considered statistically significant if it exceeded two standard deviations (2SD) of the second mode.

## 3. Results and Discussion

Consumers’ exposure to nutrition and health claims and symbols on pre-packed foods is presented in Table 2 and supplementary Table S1. We determined that 37% of the products were labeled with nutrition claims, while those products represent a share of about half the market. Consumers’ exposure to health claims is considerably lower; 13% of products were labeled with at least one health claim (SCE), representing 11% of the sales volume (SWE). The difference between the SCE and SWE values indicates that, in comparison to the food product with average sale, products with above average sale are more frequently labeled with nutrition claims (SWE/SCE ratio = 1.2) and less frequently with health claims (SWE/SCE ratio = 0.8). It should be noted that these ratios should not serve as direct indicators of the claims’ potential to influence purchasing decisions, which are affected by several different parameters, including price and brand name.

A nutrition declaration (data about nutritional composition of food) was found on 67% of the investigated products (SWE: 72%). While in some food categories almost all products were labeled with a nutrition declaration (for example within fruit juices, milk and yoghurt imitates), in some categories the penetration was very low (for example 13% and 14% on teas and eggs, respectively). According to the NHCR all pre-packed foods bearing nutrition and/or health claims should also be labeled with a nutrition declaration. We observed that some products were noncompliant with this requirement, but this does not necessarily mean that such foods are not in compliance with EU food law. This is due to the transitional measures defined in Article 28(2) of the NHCR which states that products bearing trademarks or brand names existing before 1 January 2005 which do not comply with the NHCR may continue to be marketed until 19 January 2022. However, the absence of a nutrition declaration was also observed on some foods where the nutrition and/or health claim was not part of the trademark or brand name.

Since sales weighted consumers’ exposure to different food labeling information is being reported for the first time, a comparison with other studies is not possible. However, we can compare the frequencies of specific food labeling information (SCE values), but we need to have in mind that different inclusion/exclusion food selection criteria were used in various studies. Nevertheless, notable similarities and differences can be observed if we compare the results with those from either EU or non-EU countries. Storcksdieck genannt Bonsmann *et al.* [22] reported lower penetration of nutrition claims (25%) for the overall European market (EU-27 + Turkey), but only five food categories were investigated, namely sweet biscuits, breakfast cereals, pre-packed chilled ready meals, carbonated soft drinks, and yoghurts. Lower prevalence of nutrition claims (29%) was also reported in a recent UK study, performed on a sample of foods available in an on-line store of a major food retailer [24]. However, higher penetration (47%) was observed in an Irish study [23] with a very similar design to our study (a few additional food categories were added in our study; see *Collection of data*). Similarly, a higher frequency of nutrition claims was also observed in Canada (46%) [14], in the USA (39%) [15,16] and Australia (36%) [21]. Similar trends can be observed in the relationship with health claims, where 18% prevalence was reported by Lalor *et al.* [23] (Ireland), 15% by Kaur *et al.* [24] (UK), and 2% (front-of-pack) or 4% (back-of-pack) by Storcksdieck genannt Bonsmann *et al.* [22] for the EU-27 + Turkey. Interestingly, notably lower prevalence of both nutrition and health claims (6%) was reported for Serbia [31], one of candidate countries for EU membership. Due to major regulatory differences between different jurisdictions, comparison in the use of health claims between different countries is relevant mostly for disease risk reduction claims and discussed later on.

Use of health symbols on food labels could help consumers make healthier food choices and overcome confusion in understanding food labels [32,33,34]. Therefore, we also investigated the use of *symbol of protective food*, the only health symbol found on food labels in the Slovenian market (Table 2). While the penetration of this symbol in the whole sample was relatively low (2%), higher penetration was observed for yoghurts (14%), vegetable oils (10%), and breakfast cereals (7%).

Large differences were found in the use of nutrition and health claims between different food categories. The highest penetration of nutrition claims was observed for fruit juices and flavored bottled water (95%), breakfast cereals (79%), yoghurts (65%), and milk and yoghurt imitates (94% and 81%, respectively). Yoghurts and their imitates were also commonly labeled with health claims (51% and 63%, respectively). Above-average use of health claims was also observed for breakfast cereals (31%), vegetable oils (20%), teas (19%), butter and spreads (18%), and fruit juices (15%). For comparison, we should note that yoghurts (50%), breakfast cereals (42%), butter and spreads (30%), and teas (24%) were also food categories with a high penetration of health claims in the Irish study [23], which did not cover vegetable oils. Sales weighting revealed increased exposure to claims in cheese, particularly in fresh uncured cheese, while contrary was observed in the categories eggs, bread, and vegetable oils.

Sampling of the foods for this study was done in four different food stores and it was, therefore, interesting to investigate the differences in the penetration of claims between those stores. The prevalence of nutrition and/or health claims on pre-packed foods in the large supermarket, the two neighborhood stores and the discounter is presented in Figure 1 and in supplementary Table S2. In the discounter, a significantly lower prevalence of nutrition and/or health claims was found in 17 out of 24 food categories, while in the other food stores the differences were significant only in a few food categories. Retailers are known to mostly focus on competitive price of items, rather than wide choice, and this could partially explain our observations.

To gain an insight into the type of claims used on food labels, we next identified nutrients and other substances which are most commonly mentioned in nutrition claims. Nutrition claims were most frequently related with vitamins (found on 10% of foods; sum of claims for all vitamins), minerals (8%), dietary fibre (7%), fat (4%), and sugar (4%) (Table 3; supplementary Table S3).

Vitamin claims most commonly targeted vitamin C (found on 4.7% of foods), vitamin B6 (4.4%), niacin (4.0%), thiamine (3.7%), and vitamin E (3.5%). Mineral claims were mostly related with calcium (found on 6.0% of foods). Large variations were observed between different food categories. Low energy and other energy-related claims are most common on flavored bottled water (87%), protein claims on milk/yoghurt imitates (19%–20%), salt/sodium-related claims on breads (8%), and fiber claims on breakfast cereals (38%) and breads (33%). Sugar-related claims, such as *no added sugars*, are found on 70% of fruit juices, which represent over 95% of fruit juices sold. Sugar-related claims are also quite common on breakfast cereals (15%), although the sale of such products only represents 5% of the market volume. A large penetration of low/reduced fat and other fat-related claims was found on yoghurts (23%) and their imitates (22%), spreadable vegetable fats (13%), breakfast cereals (8%) and ready meals (6%). Interestingly, while only 6% of cheese products were labeled with such fat-related claims, such products represent 24% of the cheese market (SWE/SCE ratio = 4). A similar trend was observed in spreadable vegetable fats labeled with omega-3 claims (SWE/SCE ratio = 2.4). Claims targeting minerals were mostly observed on milk (47%), milk/yoghurt imitates (34% and 41%, respectively), and breakfast cereals (37%). Breakfast cereals were also commonly labelled with vitamin claims (47%); a relatively high penetration of such claims was also observed in energy drinks (95%), isotonic and sport drinks (55%), fruit juices and nectars (31% and 27%, respectively), spreadable vegetable fats (22%, SWE: 45%), seed oils (23%), and yoghurt imitates (19%).

In addition to the above-mentioned nutrients, nutrition claims can also target other substances (see supplementary Table S3). About 38% of yoghurts (SWE: 33%) and 48% of yoghurt imitates (SWE: 48%) were labeled as containing probiotics. Phytosterols/stanols claims were found on 2% of spreadable vegetable fats (SWE: 2%). A similar penetration of these claims was also observed on milk, although the sales volume of such products represents a mere 0.2% of the market. Coenzyme Q10 and l-carnitine claims were found in 4% and 3% of yoghurts, respectively; SCE and SWE values are very similar. l-carnitine claims are also common in energy drinks (5%). However, energy drinks were mostly labeled with claims about caffeine (86%, NF: 88%) and taurine (62%; NF: 48%). Guarana claims were found on 19% of energy drinks, but these represent only a 0.4% share of the market. For comparison with other studies, fat-related nutrition claims were reported to be most common in Ireland (followed by claims related to sugars, vitamins and minerals) [23], Canada (followed by claims about vitamins and minerals) [14], the USA [15], and Australia (followed by claims about vitamins and minerals) [21].

In the next phase, we focused on analyzing the identified health claims (Table 4, supplementary Table S4). As presented in Table 1, in the EU the regulation distinguishes three different types of specific health claims: the largest category of *function claims (FC)*, *children’s development and health claims (CDHC),* and *reduction of disease risk claims (RDRC)*. In addition, foods labeled with such specific health claims can also make references to general, non-specific benefits of the nutrient or food for overall good health or health-related well-being. We found such *general non-specific health claims* on about 6% of the investigated products, either in the form of words or as pictures implying health relationships. The highest prevalence was found on yoghurts (35%) and their imitates (56%), flavored bottled water (15%) and spreadable vegetable fats (14%).

On the other hand, specific *function claims* were found on 7% of foods; the highest penetration was observed for isotonic and sport drinks (45%), yoghurts (24%) and breakfast cereals (20%). The frequency of using *CDHC* and *RDRC* was very low, only 0.1% and 0.2%, respectively. Disease reduction claims were also rarely found in the Irish study [23], while CHDC*s* were a little more common there, particularly on cheeses. The dominance of the function claims in our study can be explained by the fact that the data collection was performed before the EU Register of health claims was fully implemented [35]. When the data were being collected in 2011, FCs were in the process of scientific evaluation and their use on foods was possible without pre-approval, but pre-approval was already needed for both CDHCs and RDRCs. Function claims were added to the EU Register in 2012 and it will be interesting to examine how this has affected the food supply in the European Union. We need to mention that, contrary to the EU, in some jurisdictions pre-approval is only needed for RDRC, while function claims are less regulated [36,37,38]. This includes the case of Canada where RDRC can be found on less than 2% of foods [14].

To obtain an insight into the health relationships referred to in the health claims, the identified health claims were classified according to ICF/WHO body functions [30] (Table 4, supplementary Table S4). We determined that the claims mostly target *digestive system functions* (such claims were found on 2.8% of investigated foods), particularly in relationship with *weight maintenance* (1.4%) and *glycemic response* (0.8%). Such claims were particularly common on breakfast cereals (7% and 3%, respectively) and pasta (4% and 5%, respectively). Other food categories with a relevant penetration of weight-reduction claims include flavored bottled water (14%), yoghurt imitates (7%) and yoghurts (3%). Interestingly, slimming claims were also dominant in the Irish market and commonly found on dairy products, biscuits, bread and bakery products, teas, breakfast cereals and pasta [23]. About 2% of the foods investigated in our study were labeled with claims about immunological system functions; the highest penetration of such claims was observed in teas (9%), yoghurts (7%) and fruit juices (5%). Within other health relationships, only mental function claims were found on at least 1% of the products; food categories with the highest penetration of such claims are isotonic and sport drinks (35%) and breakfast cereals (5.8%). Claims on cardiovascular system functions were mainly dominant on seed oils (8%) and (omega-3 enriched) whole eggs (5%). Claims related to maintenance/lowering of blood cholesterol were mainly found on spreadable vegetable fats (4%), breakfast cereals (3%) and (phytosterols-enriched) milk (2%).

A systematic review [39] of studies that assessed the effect of product health information at the point of purchase on actual purchase behavior found poor evidence for such effects, but our results show that such conclusions should be taken with care. While in some EU countries the use of health claims on foods was possible even before the harmonization of legislation in 2007 (on the basis of local regulations and codes of practices), this was not the case in Slovenia. It is therefore surprising that the use of health-related information on food labeling is relatively common, indicating high interest of food manufacturers and retailers for such labeling options. The fact that nutrition and/or health claims were found on about half the volume of the pre-packed food market is a clear indication to policy-makers that this area must be carefully regulated. While it is encouraging that extensive research on the role of health-related claims and health-related symbols in consumer behavior is currently being funded [8], the European Commission also needs to assure that the existing regulation is implemented. For example, due to the danger that a nutrition or health claim might encourage excessive consumption of specific foods nutrient profiles should have been introduced in the EU in 2009, but the discussion on this stopped years ago [12]. We recently reported that over half the breakfast cereals on the Slovenian market can be classified as “less healthy” [40]; within *cereals for kids* and *Bubbles, flakes and puffs* this was the case for about three-quarters of products on the market. The results reported herein on the high penetration of health-related food information on such foods clearly support the introduction of nutrition profiles. For example, such an approach is used in New Zealand where the nutritional quality of breakfast cereals in the food supply is considerably better than in Slovenia [18]. The nutritional composition of foods in the food supply in Slovenia is therefore the subject of further investigations [41,42].

A major strength of the reported study is that it is an academic-commercial collaboration which enabled cost-effective use of sales data to determine sale-weighted consumers’ exposure to nutrition and health claims. Food retailers operate with detailed sales data on any food products available and the use of such data does not incur any additional costs. Such an approach facilitates the assessment of the penetration of food information on foods on the market in unprecedented dimensions, particularly if the partnership combines different retailers accounting for the majority market share, as was the case in this study. Further, considering that the majority of the market is included, the habits of consumers of all demographic backgrounds and from both urban and non-urban areas are well represented. The study protocol is very robust and enables changes in the food market to be efficiently controlled over time. The database compiled in such study can be used for a variety of purposes, including nutrition and public health research.

A limitation of this and other studies investigating the penetration of information on food labels lies in the use of different food categorization systems. Different categorization systems are used by different research groups, regulators and policy-makers in different countries. To assure comparability of the results with the only currently available European study on the penetration of health claims on pre-packed foods, we adopted the food classification system used in that study [23]. To make sure the study results would be usable for the government agencies and European Commission, foods were further classified according to the FOODEX2 classification system [29], which is officially used in our jurisdiction. We should note that an international collaborative project is underway within the Global Food Monitoring Group in which processed foods are surveyed using a standardized methodology in a number of countries [43]. This methodology also provides a simple food categorization system which can be used in future studies (either alone or in combination with other food categorization systems) to ensure easier comparison of the results between different studies. Although this project does not yet cover Slovenia and many other countries, it could significantly contribute to the harmonization of food classification issues.

Representativeness of the sample is also an important issue in studies which investigate the food supply. Similarly to previous studies, in this study sampling was done in grocery stores in an urban area. However, we should note that the surveyed shops were operated by retailers which operate with over 1000 grocery stores in Slovenia, in both urban and non-urban areas. 12-month sales data from shops across the whole country were used in the sales weighting approach, balancing the importance of each food in the sample. Another possible limitation of our study is the fact that data from food labels were collected directly in the food stores. Although a similar approach was used in most other studies [23,27], taking pictures of food labels provide better options for verification of results. We would also suggest that location of claims (*i.e.*, front or back of package) and price data are collected in further studies.

It should also be mentioned that collaboration and mutual trust between academia and food retailers is a precondition for using our methodology successfully and this could be a significant limitation in some environments. In our case, the trust was gained through support for our efforts from the authorities, open discussions on issues related to data sharing, and by our strict commitment to data protection. To prove this commitment to the retailers, we were the first research organization in Slovenia to have its information security management system accredited according to ISO 27001.

## 4. Conclusions

We presented a new methodological approach to assess consumers’ exposure to health-related labeling of pre-packed foods in the food supply. For the first time, sales weighting was successfully used to provide a reliable estimation of consumers’ exposure to nutrition and health claims and symbols. We determined that about half of the volume of the pre-packed food market in Slovenia is labeled with either nutrition or health claims. Large differences were found between different food categories, and also between different stores. The lowest prevalence of nutrition and health claims was found in the discounter store. The highest penetration of nutrition claims was observed on fruit juices and flavored bottled water, breakfast cereals, yoghurts and milk and yoghurt imitates. The most frequent were claims about the content of vitamins and minerals. Health claims were most commonly found on yoghurts, their imitates and breakfast cereals. While *children’s development and health claims* and *reduction of disease risk claims* were used very rarely, *general non-specific health claims* and *function claims* were found on 7% and 6% of foods, respectively. The most frequently targeted health relationships are digestive system functions (including weight maintenance and glycemic response), immunological system functions and mental functions. The reported methodology is simple and can therefore be easily repeated at a later date and/or in other countries to provide further insights into the food supply and to support the scientific community, governments and the food industry in developing further strategies to fight against food-related non-communicable diseases. Those insights will also be very valuable when planning further studies about the influence of health-related food labeling on consumers’ preferences and food choices. Such studies are currently mostly being performed using a few selected claims and such a selection can now be done considering consumers’ exposure to claims in a real-life environment. Future studies should also focus on investigating the nutritional quality of foods labeled with nutrition, health claims and symbols.

## Supplementary Materials

Supplementary materials can be accessed at: http://www.mdpi.com/2072-6643/7/11/5474/s1.
